# Grazing enhances species diversity in grassland communities

**DOI:** 10.1038/s41598-019-47635-1

**Published:** 2019-08-01

**Authors:** Muhammad Almaududi Pulungan, Shota Suzuki, Maica Krizna Areja Gavina, Jerrold M. Tubay, Hiromu Ito, Momoka Nii, Genki Ichinose, Takuya Okabe, Atsushi Ishida, Masae Shiyomi, Tatsuya Togashi, Jin Yoshimura, Satoru Morita

**Affiliations:** 1 Graduate School of Science and Technology and Department of Mathematical and Systems Engineering, Shizuoka University, 3-5-1 Johoku, Naka-ku, Hamamatsu, 432-8561 Japan; 2 Mathematics Division, Institute of Mathematical Sciences and Physics, University of the Philippines Los Baños, College, Laguna, 4031 Philippines; 3 Department of International Health, Institute of Tropical Medicine, Nagasaki University, Nagasaki, 852-8523 Japan; 4 Department of Environmental Sciences, Zoology, University of Basel, Basel, 4051 Switzerland; 5 Graduate School of Integrated Science and Technology, Shizuoka University, 3-5-1 Johoku, Hamamatsu, 432-8561 Japan; 6 Center for Ecological Research, Kyoto University, Otsu, Shiga 520-2113 Japan; 7 Faculty of Science, Ibaraki University, 2-1-1 Bunkyo, Mito, Ibaraki 310-8512 Japan; 8 Department of Environmental and Forest Biology, State University of New York College of Environmental Science and Forestry, Syracuse, NY 13210 USA; 9 Marine Biosystems Research Center, Chiba University, Kamogawa, Chiba 299-5502 Japan

**Keywords:** Biodiversity, Community ecology, Grassland ecology, Theoretical ecology, Ecological modelling

## Abstract

In grassland studies, an intermediate level of grazing often results in the highest species diversity. Although a few hypotheses have been proposed to explain this unimodal response of species diversity to grazing intensity, no convincing explanation has been provided. Here, we build a lattice model of a grassland community comprising multiple species with various levels of grazing. We analyze the relationship between grazing and plant diversity in grasslands under variable intensities of grazing pressure. The highest species diversity is observed at an intermediate grazing intensity. Grazers suppress domination by the most superior species in birth rate, resulting in the coexistence of inferior species. This unimodal grazing effect disappears with the introduction of a small amount of nongrazing natural mortality. Unimodal patterns of species diversity may be limited to the case where grazers are the principal source of natural mortality.

## Introduction

Grasslands are a vital part of the world’s ecosystems and occupy more than one-quarter of Earth’s land area^1^. Some grasslands contain more species than tropical forests at a small scale, e.g., 15.5–19.7 species in a 0.01 m^2^ area of alpine meadow on the Qinghai-Tibet plateau^2^, 43 species in a 0.1 m^2^ area of Romanian grassland^3^, and a new world record with 17 species in 0.0044 m^2^ of Krkonoše Mountains^4^. Maintaining the species diversity of grasslands is crucial for avoiding productivity loss, which in turn affects ecosystem processes^5,6^.

Many previous studies sought to explain the factors influencing the diversity of grasslands. Resource partitioning plays a significant role in promoting diversity^7,8^. Seed dispersal is also an important factor affecting the coexistence of multiple plant species^9–11^. Another factor that positively affects diversity is environmental heterogeneity, which exists in all natural systems, including grasslands^12–14^. However, all these studies concluded a role of niche differentiation or niche partitioning in determining species diversity without explicitly considering the role of grazing.

Many studies of natural, seminatural and managed grasslands have shown that an intermediate level of grazing results in the highest species diversity. Excessive grazing reduces species diversity because it converts grassland to bare land. However, why the species diversity of grasslands increases with the introduction of/an increase in grazing to some point is unclear. Based on previous empirical studies, the introduction of light grazing or an increase in grazing intensity increases the species diversity in some grasslands. For example, in five types of grassland communities in Andorran mountains, the highest plant diversity and resulting functional richness were observed at the intermediate grazing intensity^15^. In rangelands located in Tibetan alpine meadows, light and moderate grazing promote biodiversity and increase nectar volume at both the individual level and the community level^16^. In Himalayan rangelands, moderate grazing increases diversity^17^. Heavy grazing indirectly influences species richness in Inner Mongolian grassland sites^18^. Thus, explanations of why and how an increase in grazing intensity promotes the coexistence of many more species are needed.

Previous studies have modeled the coexistence of vegetation. The lottery model revealed the importance of environmental variability in promoting coexistence^19–21^. However, lottery models eventually lead to competitive exclusion over a very long time scale^22^. Therefore, a lottery model is not appropriate for testing the effects of grazing because of the eventual extinction of all but one species. We need a spatial component in the model to achieve the coexistence of plant species as in lattice models^22^.

Using a lattice model, Kakishima *et al*. examined the contribution of animal seed dispersers to the coexistence of tree species^23^. Tubay *et al*. applied this lattice model to build a general model of plant communities and reported the importance of spatial heterogeneity in determining the coexistence of many species^22^. These two models are able to avoid competitive exclusion as a stable state. Therefore, we can test the effects of grazing by using these lattice models. Here, we introduce grazing effects into a lattice model of a grassland community to examine how species diversity is affected by grazing intensity. We modify the lattice community model of Tubay *et al*. to develop a two-layer lattice model of a grassland community under grazing (with an animal layer)^22^. Using our model, we examine the effects of animal grazing on grasslands and investigate the underlying mechanism of these effects. The highest species diversity in the grasslands is observed under an intermediate grazing intensity. In contrast, species diversity decreases with an increase in grazer density. Furthermore, this unimodal grazing effect disappears with the introduction of a small amount of nongrazing natural mortality. We discuss when, why and how the highest diversity is achieved at an intermediate grazing intensity. We also discuss the implications of our results for the management of grassland communities.

## Results

**Figure 1:**
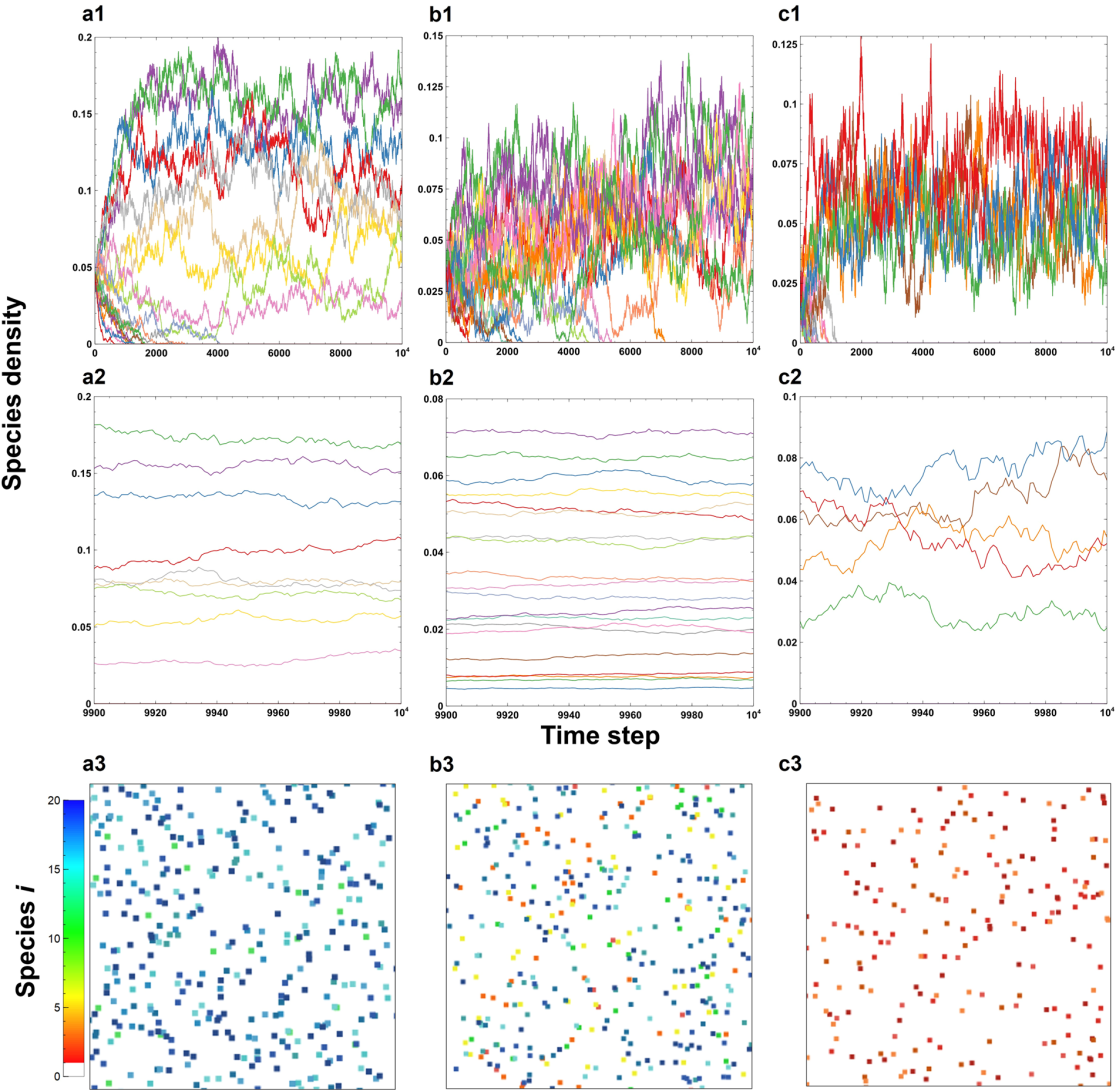
The temporal dynamics of species density and a snapshot of the final density composition. **a1**-**c3** The grazing intensity of a species decreases as the initial birth rate decreases, i.e., ${g}_{i}=G+G^{\prime} (20-i)$ and *B*_*i*_ = *B*−0.002(*i*-1) for *i* = 1, 2, …, *s*), indicating a trade-off between the birth rate and grazing intensity of a species. **a2**, **b2**, **c2** Temporal dynamics of the last one hundred steps. **a3**, **b3**, **c3** A snapshot of the final surviving species composition. The value of the species-dependent factor is *G*′ = 0.0005. The basal grazing intensities are (**a1**, **b1**, **c1**) *G* = 0.05, (**a2**, **b2**, **c2**) *G* = 0.15 and (**a3**, **b3**, **c3**) *G* = 0.4. The lattice size is 100 × 100. The dispersal distance is *P* = 40. The initial species density is *I*_*i*_ = 0.03 (same for all species). The density of grazer cells is *I*_*y*_ = 0.4. Error bars indicate the standard deviations.

First, we simulate a lattice grassland model with 20 plant species under various levels of grazing intensity (*G*) without nongrazing natural mortality, i.e., *d*_*i*_ = 0 (Fig. 1, Supplementary Figure 1). The temporal dynamics exhibit large fluctuations in the number of individuals of each species over a long period of time (Fig. 1a1,b1,c1). In contrast, this number is fairly stable over a short timescale (Fig. 1a2,b2,c2). Based on the short-term temporal dynamics (Fig. 1a2,b2,c2) and snapshots of the plant distributions (Fig. 1a3,b3,c3), the number of surviving plant species is highest at an intermediate grazing intensity (*G*).

**Figure 2:**
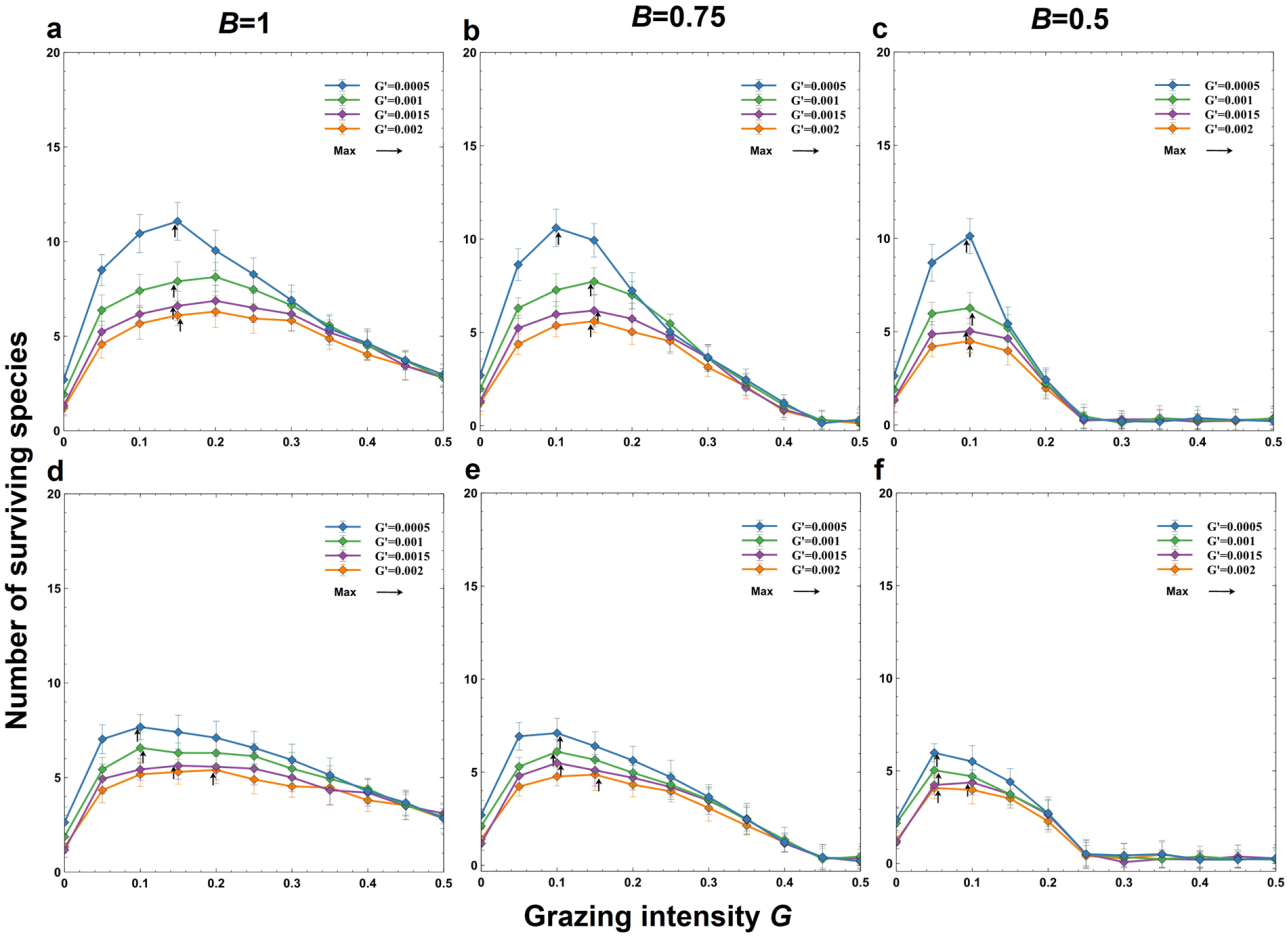
The number of surviving species as a function of the basal grazing intensity *G* for various values of interspecific grazing differences (*G*′). Three different values of the basal birth rate are shown: (**a**,**d**) *B* = 1; (**b**,**e**) *B* = 0.75; and (**c**,**f**) *B* = 0.5. (**a–c**) The grazing intensity of a species decreases as the initial birth rate decreases, i.e., ${g}_{i}=G+G^{\prime} (20-i)$ and *B*_*i*_ = *B*−0.002(*i*-1) for *i* = 1, 2, …, *s*), indicating a trade-off between the birth rate and grazing intensity of a species. (**d**–**f**) The grazing intensity increases as the initial birth rate decreases, i.e., ${g}_{i}=G+G^{\prime} (i-1)$ and *B*_*i*_ = *B*−0.002(*i*-1) for *i* = 1, 2, …, *s*), indicating that inferior species are eaten more often by grazers. The lattice size is 100 × 100. The dispersal distance is *P* = 40. The initial species density is *I*_*i*_ = 0.03 (same for all species). The density of grazer cells is *I*_*y*_ = 0.4. Error bars indicate the standard deviations.

We vary the basal grazing intensity *G* to examine the effects of grazing (Fig. 2, Supplementary Figures 3, 4, Supplementary Table 1). The number of species is highest at an intermediate grazing intensity (*G*) for a given fecundity *B* and interspecific grazing difference *G*′ (Fig. 2; Supplementary Figure 2b,c,e,f). The interspecific grazing difference *G*′ is the difference in animal preference or selectiveness in grazing, e.g., palatable herbs or unpalatable grass. To examine the effects of a trade-off between birth rate and grazing intensity, we consider the following opposite relations between the species-specific expected birth rate *B*_*i*_ and grazing rate *g*_*i*_: (1) the trade-off case: species with a high *B*_*i*_ have a high *g*_*i*_ (grazing is subject to a trade-off) and (2) the opposite case: species with a high *B*_*i*_ have a low *g*_*i*_. In both cases, the number of surviving species exhibits unimodal behavior in response to the grazing intensity *G*. The number of coexisting species at peak diversity is larger under the first case (with the trade-off) than under the second case (the opposite) (Fig. 2).

**Figure 3:**
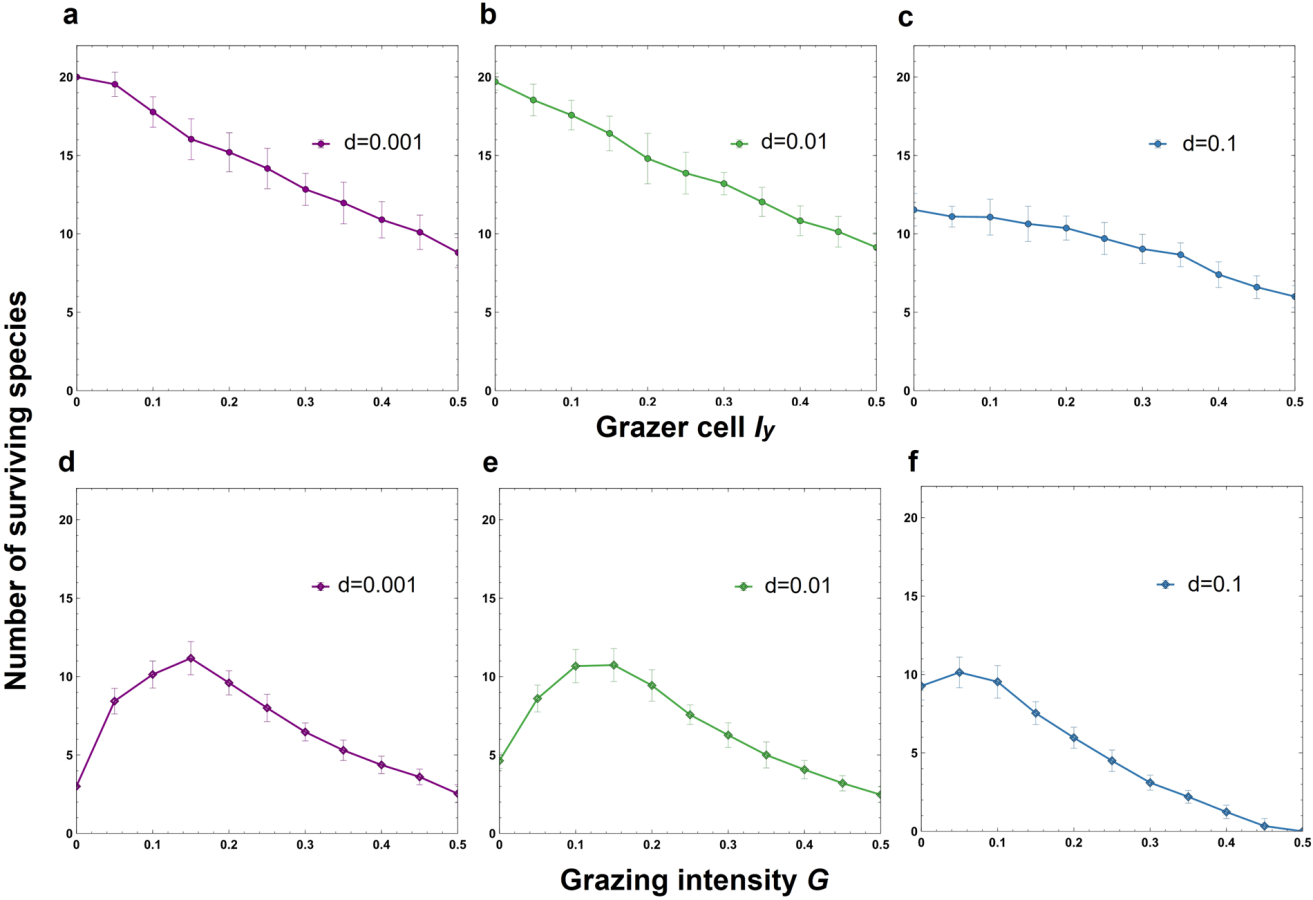
The effects of the animal density *I*_*y*_ on the number of surviving species under various basal grazing intensities (*G*) and natural mortalities (*d*_*i*_). (**a**–**c**) The grazing intensity of a species decreases as the fecundity decreases, i.e., ${g}_{i}=G+G^{\prime} (20-i)$ and *B*_*i*_ = *B*−0.002(*i*-1) for *i* = 1, 2, …, *s*), indicating a trade-off between the birth rate and grazing intensity of a species. The lattice size is 100 × 100. (**a**–**c**) The grazing intensity is 0.15. The dispersal distance is *P* = 40. The initial species density is *I*_*i*_ = 0.03 (same for all species). (**d**–**f**) The density of grazer cells is *I*_*y*_  = 0.4. Error bars indicate the standard deviations.

If there is no interspecific grazing difference (*G*′ = 0), then the number of surviving species decreases monotonically with an increase in the grazing intensity *G* (Supplementary Figures 2a,d, 3a, 4a,c). However, if a slight difference (e.g., *G*′ = 0.0005) is introduced, then the number of species responds unimodally (Fig. 2, Supplementary Figures 2b,c,e,f, 3b, 4b,d). Interestingly, the peak height (the number of surviving species) decreases as the interspecific grazing difference *G*′ is further increased (differences in colored lines in Fig. 2, Supplementary Figures 3b,c). The number of coexisting species decreases as the density of grazer cells *I*_*y*_ increases (Fig. 3a–c; Supplementary Figure 4a). However, the unimodal behavior is preserved as long as the density of grazer cells is kept constant (Fig. 3d–f; Supplementary Figure 4b,d). Natural mortality *d*_*i*_ (i.e., *d*_*i*_ = 0.1) also suppresses the unimodal behavior (Fig. 3f).

**Figure 4:**
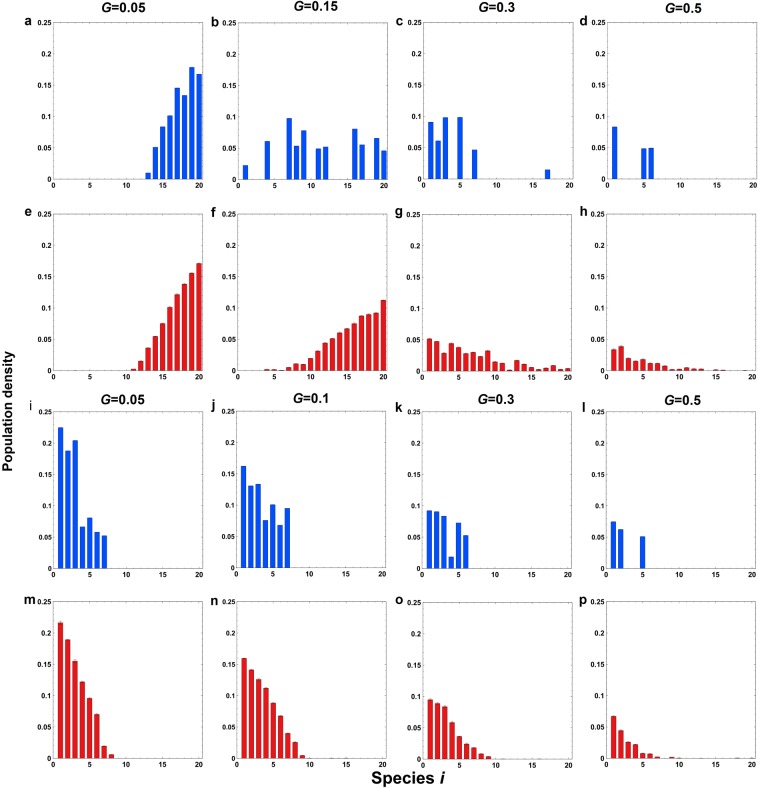
Population densities of species with respect to the basal grazing intensity *G*. (**a**–**h**) The grazing intensity of a species increases as the fecundity increases, i.e., ${g}_{i}=G+G^{\prime} (20-i)$ and *B*_*i*_ = *B*−0.002(*i*-1) for *i* = 1, 2, …, *s*), indicating a trade-off between the birth rate and grazing intensity of a species. (**i**–**p**) The grazing intensity decreases as the fecundity increases, i.e., ${g}_{i}=G+G^{\prime} (i-1)$ and *B*_*i*_ = *B*−0.002(*i*-1) for *i* = 1, 2, …, *s*), indicating that inferior species are eaten more often by grazers. (**a**–**d**,**i**–**l**) The result of a single run. (**e**–**h**,**m**–**p**) The average of 30 runs. The basal grazing rates are (**a**,**e**,**i**,**m**) *G* = 0.05, (**b**,**f**,**j**,**n**) *G* = 0.1, (**c**,**g**,**k**,**o**) *G* = 0.3, and (**d**,**h**,**l**,**p**) *G* = 0.5. The parameters are as follows: interspecific grazing difference, *G*′ = 0.0005; basal fecundity, *B* = 1; initial species density, *I*_*i*_ = 0.03 (for all species); density of grazer cells, *I*_*y*_ = 0.4; and dispersal distance, *P* = 40. The lattice size is 100 × 100.

To understand the effect of grazing on population density, we examine the composition of surviving species (Fig. 4). In the first (trade-off) case, the species with the lowest grazing rate dominates at a low grazing intensity (Fig. 1a3), and that with the highest birth rate dominates at a high grazing intensity (Fig. 1c3), while various species survive at low densities at an intermediate grazing intensity (Fig. 1b3). In contrast to the second case (opposite to the trade-off case) (Fig. 4i–p), however, the results of the first case (with the trade-off) reveal a large fluctuation when the grazing intensity *G* is high (Fig. 4a–h). Here, which species survive depends on the simulation run (Fig. 4c,d,g,h). In any case, the results converge, and monotonic behavior is recovered as *G*′ decreases to zero (Supplementary Figure 2a,d). Accordingly, the results indicate that the species dependence of grazing *G*′ is important for unimodality but the trade-off between the birth rate and grazing intensity is not essential.

We also examine the effects of dispersal distance and lattice size on the number of surviving species (Supplementary Figure 5). We find no effects of dispersal distance on surviving species (Supplementary Figure 5a). Furthermore, the number of surviving species increases with the lattice size (Supplementary Figure 5b).

## Discussion

The current report demonstrates that the empirically observed promotion of diversity by grazing is possible. This paper presents the first possible mechanism to explain the enhancement of species diversity by grazing. Our result confirms that the coexistence of plant species is possible under grazing in grasslands (Fig. 1). The results reveal unimodality in which the peak species diversity occurs at an intermediate grazing intensity (Fig. 2). When there is a trade-off between the birth rate and grazing rate, the number of coexisting species is large (more than 10 species) (Fig. 2a–c). Therefore, this trade-off promotes coexistence by balancing competitiveness among all plant species. We also test the case in which the grazing rate is negatively correlated with the birth rate. Surprisingly, unimodality in response to grazing intensity is still observed (Fig. 2d–f). In this case, the intermediate grazing rate allows less superior species to persist by occupying the new vacant sites produced by grazers (Fig. 4j,n), while it is not strong enough to exclude them (as in Fig. 4l,p). Thus, the coexistence of species is mechanically promoted because (weak) grazing produces vacant sites available for other species. The number and composition of coexisting species vary depending on simulation runs (Fig. 4a–d,i–l), while on average they are determined by the birth rate and grazing mortality rate (Fig. 4e–h,m–p). The trade-off between the birth rate and grazing rate is not necessary for the unimodality in response to grazing intensity.

The promotion of coexistence by grazing disappears when nongrazing mortality is introduced (Fig. 3d–f, Supplementary Figures 2a, 2d, 3). Therefore, an increase in diversity due to grazing does not occur when grazing is not the principal source of mortality in grasslands, i.e., when the nongrazing mortality is not negligible. This prediction should be tested empirically in future field studies. The interspecific grazing difference *G*′ also plays an important role in the promotion of coexistence. If the difference is absent or very minute, then the promotion of coexistence by grazing disappears (Supplementary Figure 3a vs Supplementary Figure 3b,c).

Which species survive under grazing depends on the interspecific relationship between the birth rate and grazing mortality rate (Fig. 4). In the trade-off case, inferior species (with low birth rates) exclude superior species (with high birth rates) (Fig. 2a–c). In the opposite case, superior species dominate the grassland (Fig. 2d–f). The composition of surviving species is highly variable in the trade-off case but stable in the opposite case (Fig. 4). These results should be verified empirically in managed grasslands. Furthermore, the current simplified assumptions about grazing rate *g*_*i*_ may be modified explicitly to include the behavior of grazing animals, e.g., the frequency dependence in food-plant selection.

The effects of herbivores on plant diversity in grasslands have been analyzed by using empirical data^24–26^. The proposed grazing model integrated with a microhabitat locality model^22^ should be sufficient as a canonical model with which to investigate the functional mechanisms of herbivore grazing effects mathematically. In terms of maintaining and increasing diversity, our results are in line with those of some empirical studies^2,15–18,27,28^. For example, Komac *et al*. stated that maintaining an adequate grazing intensity (avoiding both abandonment and overgrazing) is necessary to preserve diversity in grasslands^15^. Mu *et al*. also found that light and moderate grazing promote plant biodiversity^16^. In addition, Chen *et al*. showed that heavy grazing has an indirect influence on biodiversity^18^. Furthermore, Chen *et al*. based on empirical observation inferred that grazing type and vegetation structure that affect spatial variation are the reasons for the high species richness in the Qinghai alpine meadow^2^. The two reasons are in line with our models and results. Moreover, by modifying the lattice setting, the current model can be applied to other terrestrial communities while considering predators in the system. However, this mechanism of grazer or predator effects should be directly verified by using empirical data in the future.

We should also note that this grazing model presents similar results with the intermediate disturbance models^13,19,20,29,30^. In these models, there may be several specific underlying mechanisms promoting coexistence. Qualitatively the current grazing model appears to conform to these models. However, whether the current model is considered as one such specific mechanism or not is a future issue.

## Methods

### Lattice Model

Following the lattice models of Tubay *et al*.^22^, we analyzed a modified lattice Lotka-Volterra (LV) competition model of a grassland community with grazing:

1 $${X}_{i}+O\to {X}_{i}+{X}_{i},\,\,rate:{b}_{i}$$

2 $${X}_{i}\to O,\,\,rate:{d}_{i}$$

3 $$Y+{X}_{i}\to Y+O,\,rate:{g}_{i}$$

The parameters *b*_*i*_, *d*_*i*_ and *g*_*i*_ denote the birth rate, nongrazing death rate and grazing mortality rate of plant species *i*, respectively. The model of Tubay *et al*. represents only the situation where plant species compete for space (i.e., direct sunlight and soil)^22^. In the modified model, we add another two-dimensional lattice (100×100) of grazers that randomly feed on plants. The grazer site *Y*, i.e., the site occupied by a population of grazers, moves randomly to feed on plants. Death by grazing (Eq. (3)) occurs only in site *Y*. The density *I*_*y*_ of cells occupied by grazers (*Y*) and the initial density *I*_*i*_ of plant cells (*X*_*i*_) are kept constant. The stronger the grazing intensity is, the higher the *g*_*i*_ is. One site is either vacant or occupied by a single plant species. In contrast, one grazer site may represent multiple grazing animals, depending on the grazing intensity. Nongrazing mortality is assumed negligible, i.e., *d*_*i*_ = 0, unless otherwise stated (Fig. 3).

Following Tubay *et al*.^22^, we assume that the birth rate is given by *b*_*i*_ = *B*_*i*_ *ɛ*_*i*_[*m*,*n*], where *ɛ*_*i*_[*m*,*n*] is a random number between 0 and 1 that specifically depends both on species *i* and site [*m*,*n*], and the range of seed dispersal (dispersal distance: *P*) is kept constant, i.e., *P* = 40, unless specified. Depending on species *i*, the expected birth rate *B*_*i*_ is given by *B*_*i*_ = *B*−0.002(*i*-1) (*i* = 1, 2, …, 20), i.e., species 1 (20) is the strongest (weakest) among 20 species.

We consider the following two cases of species dependence of the grazing mortality *g*_*i*_:

Trade-off case: ${g}_{i}=G+G^{\prime} (20-i)$.

Opposite case: $${g}_{i}=G+G^{\prime} (i-1)$$.

The parameters *G* and *G*′ are the (basal, species-independent) grazing intensity and the species-dependent factor, respectively. In the first (second) case, the grazing intensity is strongest for the weakest (strongest) of 20 species.

We analyze the effect of grazing mortality (*g*_*i*_) on the number of surviving plant species according to the following simulation procedure:

1. Initial distributions
  - Individuals of plant species *i* are randomly distributed over the square-lattice cells with the initial density *I*_*i*_ ( = 0.03).
  - Grazer cells are also randomly distributed over the square-lattice cells with a given density *I*_*y*_.
2. Reaction dynamics
  - Birth process (Eq. (1)): Two cells are chosen randomly. If the cells are *X*_*i*_ and *O*, then cell *O* is changed to *X*_*i*_ with probability *b*_*i*_. Otherwise, the cells remain unchanged.
  - Death process (Eq. (2)): One cell is chosen randomly. If the cell is *X*_*i*_, then it is changed to *O* with probability *d*_*i*_. Note that this process is omitted when *d*_*i*_ = 0.
  - Grazing process (Eq. (3)): One cell is chosen randomly. If the cell is occupied by a grazer (*Y*) and species *i* (*X*_*i*_), then *X*_*i*_ is changed to *O* with probability *g*_*i*_. Otherwise, the cell remains unchanged.
3. Repetition process
  - Steps 2a-2b and 2c are repeated *L*×*L* and *k*×*L*×*L* times, respectively, where *L*×*L* is the total number of square-lattice sites and the number of grazing episodes is fixed at *k* = 3. As a default, we set *L* = 100. This whole process is called a Monte Carlo step.
4. Process 3 is repeated for a specific number (e.g., 10,000 times) of Monte Carlo steps.

## Supplementary information

Supplementary Information

Supplementary Information (code)

## Data Availability

Simulation program is provided in Supplementary Information. All data are derived from the simulation program.

## References

[1] Loveland TR (2000). Development of a global land cover characteristics database and IGBP DISCover from 1 km AVHRR data. Int. J. Remote Sens..

[2] Chen J (2007). Small-scale species richness and its spatial variation in an alpine meadow on the Qinghai-Tibet Plateau. Ecol. Res..

[3] Wilson JB, Peet RK, Dengler J, Pärtel M (2012). Plant species richness: The world records. J. Veg. Sci..

[4] Chytrý M (2015). The most species-rich plant communities in the Czech Republic and Slovakia (with new world records). Preslia.

[5] Hector A (1999). Plant Diversity and Productivity Experiments in European Grasslands. Science.

[6] Tilman D (1997). The Influence of Functional Diversity and Composition on Ecosystem Processes. Science.

[7] Sala OE, Golluscio RA, Lauentroth WK, Soriano A (1989). Resource partitioning between shrubs and grasses in the Patagonian steppe. Oecologia.

[8] McKane RB (2002). Resource-based niches provide a basis for plant species diversity and dominance in arctic tundra. Nature.

[9] Collins SL, Uno GE (1985). Seed Predation, Seed Dispersal, and Disturbance in Grasslands: A Comment. Am. Nat..

[10] Vandvik V, Goldberg DE (2006). Sources of diversity in a grassland metacommunity: quantifying the contribution of dispersal to species richness. Am. Nat..

[11] Pinto SM, Pearson DE, Maron JL (2014). Seed dispersal is more limiting to native grassland diversity than competition or seed predation. Journal of Ecology.

[12] Tilman, D. *Resource Competition and Community Structure* (Princeton Univ. Press, Princeton, New Jersey, 1982). 7162524

[13] Chesson P (2000). Mechanisms of maintenance of species diversity. Annu. Rev. Ecol. Syst..

[14] Maestre FT, Reynolds JF (2006). Spatial heterogeneity in soil nutrient supply modulates nutrient and biomass responses to multiple global change drivers in model grassland communities. Glob. Change Biol..

[15] Komac B, Pladevall C, Doménech M, Fanlo R (2015). Functional diversity and grazing intensity in sub-alpine and alpine grasslands in Andorra. Appl. Veg. Sci..

[16] Mu J, Zeng Y, Wu Q, Niklas KJ, Niu K (2016). Traditional grazing regimes promote biodiversity and increase nectar production in Tibetan alpine meadows. Agr. Ecosyst. Environ..

[17] Niu K, He J, Zhang S, Lechowicz MJ (2016). Tradeoffs between forage quality and soil fertility: Lessons from Himalayan rangelands. Agr. Ecosyst. Environ..

[18] Chen J, Shiyomi M, Wuyunna, Hori Y, Yamamura Y (2014). Vegetation and its spatial pattern analysis on salinized grasslands in the semiarid Inner Mongolia steppe. Grassl. Sci..

[19] Chesson PL, Warner RR (1981). Environmental Variability Promotes Coexistence in Lottery Competitive Systems. Am. Nat..

[20] Snyder RE, Chesson P (2003). Local dispersal can facilitate coexistence in the presence of permanent spatial heterogeneity. Ecol. Lett..

[21] Takenaka A (2006). Dynamics of seedling populations and tree species coexistence in a forest: a simulation study. Ecol. Res..

[22] Tubay JM (2015). Microhabitat locality allows multi-species coexistence in terrestrial plant communities. Sci. Rep..

[23] Kakishima S (2015). The contribution of seed dispersers to tree species diversity in tropical rainforests. R. Soc. Open Sci..

[24] Olff H, Ritchie ME (1998). Effects of herbivores on grassland plant diversity. Tree.

[25] Bakker ES, Ritchie ME, Olff H, Milchunas DG, Knops JMH (2006). Herbivore impact on grassland plant diversity depends on habitat productivity and herbivore size. Ecol. Lett..

[26] Liu J (2015). Impacts of grazing by different large herbivores in grassland depend on plant species diversity. J. Appl. Ecol..

[27] Collins SL, Knapp AK, Briggs JM, Blair JM, Steinauer EM (1998). Modulation of diversity by grazing and mowing in native tallgrass prairie. Science.

[28] Xiong D, Shi P, Zhang X, Zou CB (2016). Effects of grazing exclusion on carbon sequestration and plant diversity in grasslands of China—A meta-analysis. Ecol. Eng..

[29] Chesson P, Huntly N (1997). The Roles of Harsh and Fluctuating Conditions in the Dynamics of Ecological Communities. Am. Nat..

[30] Wilkinson DM (1999). The Disturbing History of Intermediate Disturbance. Oikos.

