# Author Correction: Grazing enhances species diversity in grassland communities

**DOI:** 10.1038/s41598-020-69594-8

**Published:** 2020-07-22

**Authors:** Muhammad Almaududi Pulungan, Shota Suzuki, Maica Krizna Areja Gavina, Jerrold M. Tubay, Hiromu Ito, Momoka Nii, Genki Ichinose, Takuya Okabe, Atsushi Ishida, Masae Shiyomi, Tatsuya Togashi, Jin Yoshimura, Satoru Morita

**Affiliations:** 1 Graduate School of Science and Technology and Department of Mathematical and Systems Engineering, Shizuoka University, 3-5-1 Johoku, Naka-ku, Hamamatsu, 432-8561 Japan; 2 Mathematics Division, Institute of Mathematical Sciences and Physics, University of the Philippines Los Baños, College, Laguna, 4031 Philippines; 3 Department of International Health, Institute of Tropical Medicine, Nagasaki University, Nagasaki, 852-8523 Japan; 4 Department of Environmental Sciences, Zoology, University of Basel, Basel, 4051 Switzerland; 5 Graduate School of Integrated Science and Technology, Shizuoka University, 3-5-1 Johoku, Hamamatsu, 432-8561 Japan; 6 Center for Ecological Research, Kyoto University, Otsu, Shiga 520-2113 Japan; 7 Faculty of Science, Ibaraki University, 2-1-1 Bunkyo, Mito, Ibaraki 310-8512 Japan; 8 Department of Environmental and Forest Biology, State University of New York College of Environmental Science and Forestry, Syracuse, NY 13210 USA; 9 Marine Biosystems Research Center, Chiba University, Kamogawa, Chiba 299-5502 Japan

Correction to: *Scientific Reports* 10.1038/s41598-019-47635-1, published online 01 August 2019

This Article contains errors.

In the Methods, under the Lattice Model subheading, the sentence,

 "In the first (second) case, the grazing intensity is strongest for the weakest (strongest) of 20 species.”

should read:

“In the first (second) case, the grazing intensity is strongest for the strongest (weakest) of 20 species."

In Supplementary Figure 4a, the data for G = 0.5 was not included. In Supplementary Figure 4b, the data was plotted incorrectly at G = 0.5, and error bars were not included. The correct Supplementary Figure 4 appears below as Figure 1.

**Figure 1:**
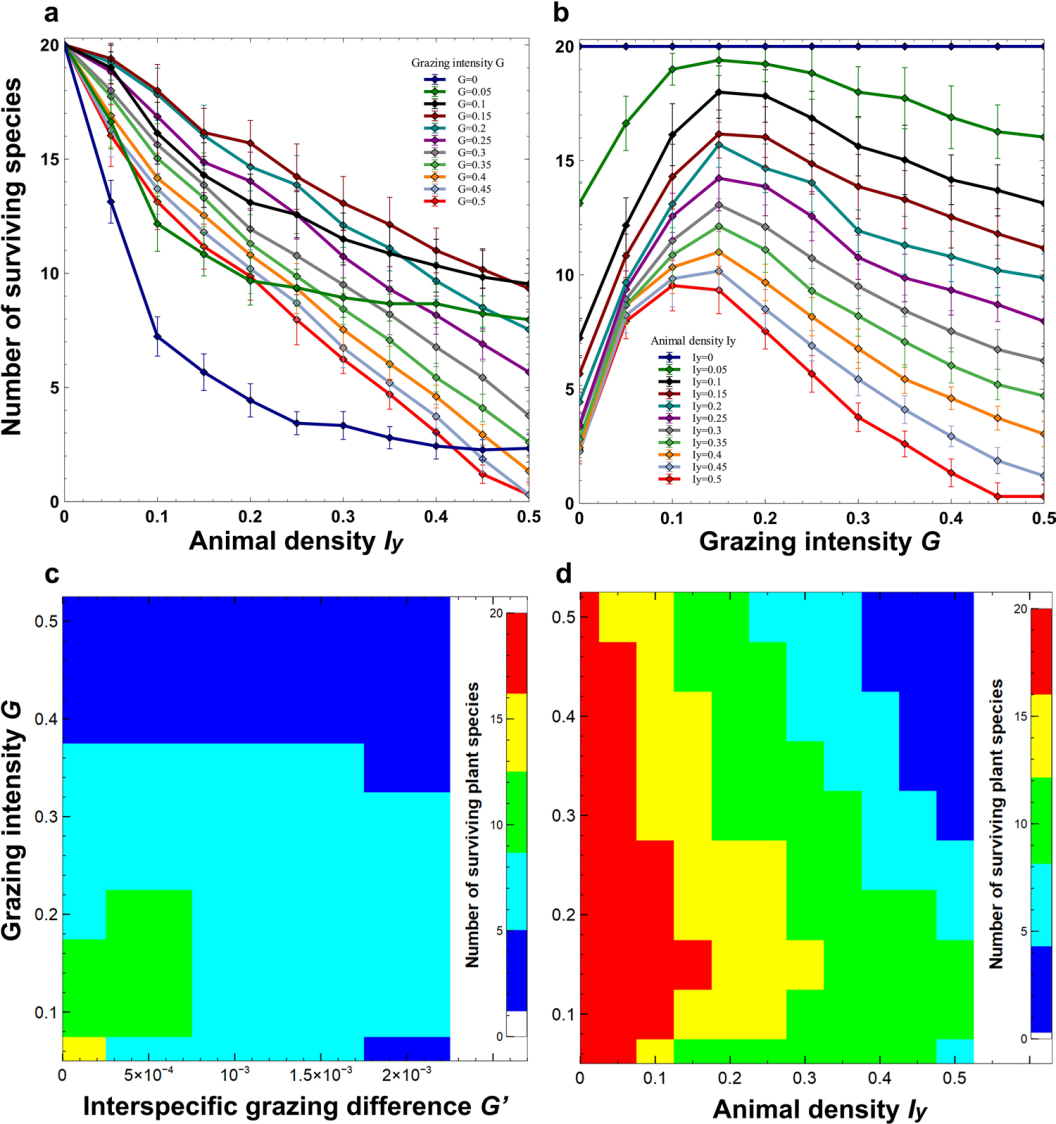
.

