# Supplementary material for: Grazing enhances species diversity in grassland communities
Source: Sci Rep. 2019 Aug 1;9:11201. doi: 10.1038/s41598-019-47635-1 (PMC6671982; doi:10.1038/s41598-019-47635-1)
Supplement: Supplementary file 1 — Supplementary Information [file 41598_2019_47635_MOESM1_ESM.pdf]

SUPPLEMENTARY MATERIALS

Title: Grazing enhances species diversity in grassland communities
Short title: **Grazing enhances biodiversity in grasslands**

Muhammad Almaududi Pulungan[1,*], Shota Suzuki[5], Maica Krizna Areja Gavina[1,2], Jerrold M. Tubay[2], Hiromu Ito[3,4], Momoka Nii[5], Genki Ichinose[1], Takuya Okabe[5], Atsushi Ishida[6], Masae Shiyomi[7], Tatsuya Togashi[9], JinYoshimura[1,8,9, *], Satoru Morita[1,*]

Contents:
Supplementary Figures 1-5
Supplementary Table 1

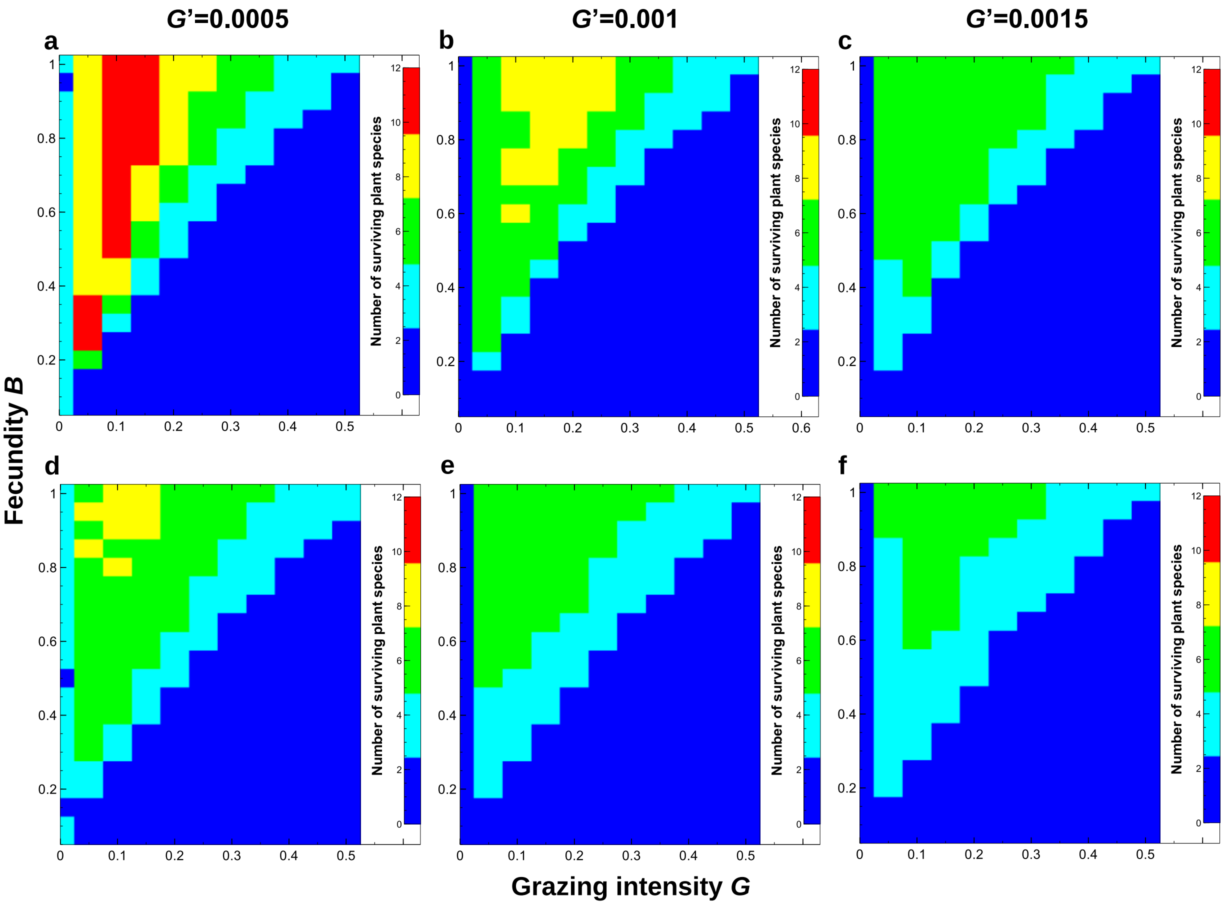

Supplementary Figure 1. The average number of surviving species as a function of the basal grazing intensity *G* and basal fecundity *B* for three values of interspecific grazing differences (*G'*). **a-c** The grazing intensity of a species increases as the fecundity increases, i.e., $g_i = G + G'(20 - i)$ and $B_i = B - 0.002(i-1)$ for i=1, 2, …, s), indicating a trade-off between the birth rate and grazing intensity of a species. **d-f** The grazing rate decreases as the fecundity increases, i.e., $g_i = G + G'(i-1)$ and $B_i = B - 0.002(i-1)$ for i=1, 2, …, s), indicating that inferior species are eaten more often by grazers. The values of interspecific grazing differences are (**a, d**) G'= 0.0005, (**b, e**) G'= 0.001, and (**c, f**) G'= 0.0015. The lattice size is 100 x100. The dispersal distance is P = 40. The initial species density is I_i = 0.03 (same for all species). The density of grazer cells is I_y = 0.4.

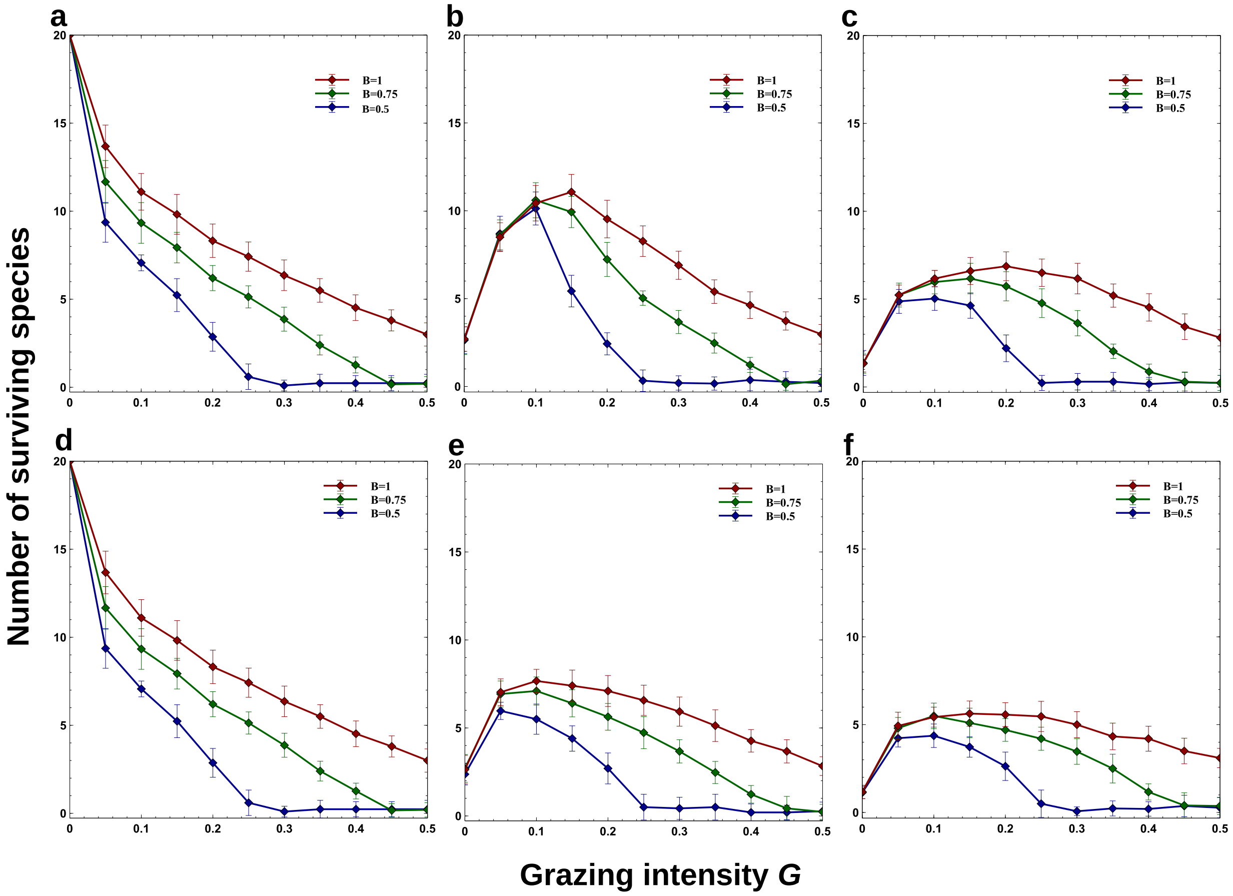

Supplementary Figure 2. The number of surviving species as a function of the basal grazing intensity G for various values of the basal birth rate B with $G'=0$. **a-c** The grazing intensity of a species increases as the fecundity increases, i.e., $g_i = G + G'(20-i)$ and $B_i = B$-0.002(i-1) for i=1, 2, …, s), indicating a trade-off between the birth rate and grazing intensity of a species. **d-f** The grazing intensity decreases as the fecundity increases, i.e., $g_i = G + G'(i-1)$ and $B_i = B$-0.002(i-1) for i=1, 2, …, s), indicating that inferior species are eaten more often by grazers. The differences in the interspecific grazing rate are (**a, d**) G'= 0, (**b, d**) G'= 0.0005, and (**c, f**) G'= 0.0015. The lattice size is 100 x100. The dispersal distance is P = 40. The initial species density is I_i = 0.03 (same for all species). The density of grazer cells is I_y = 0.4. Error bars indicate the standard deviations.

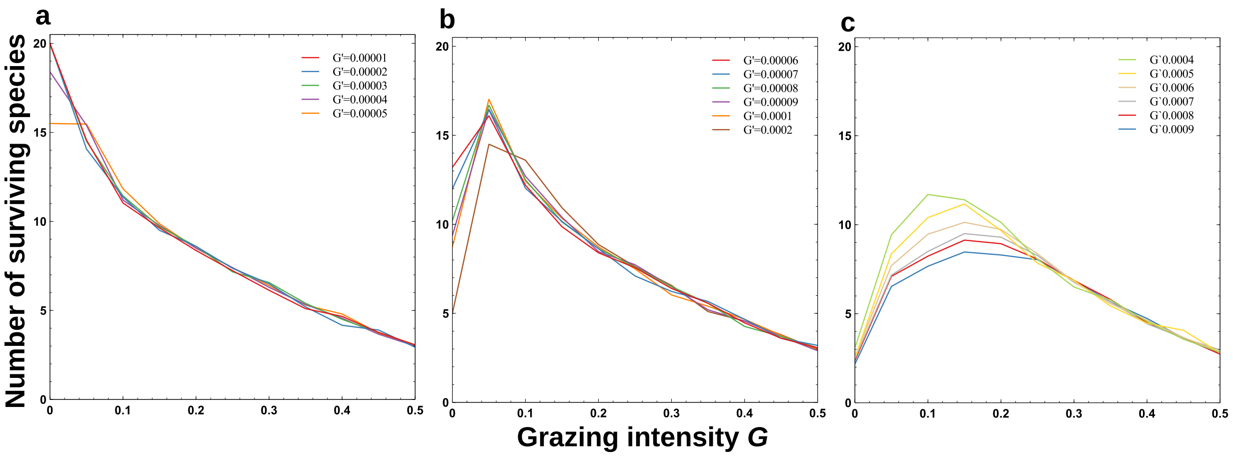

Supplementary Figure 3. The effect of the interspecific grazing difference value *G'* on the number of surviving species. **a-c** The grazing intensity of a species increases as the fecundity increases, i.e., $g_i = G + G'(20 - i)$ and $B_i = B\text{-}0.002(i\text{-}1)$ for i=1, 2, …, s), indicating a trade-off between the birth rate and grazing intensity of a species. The lattice size is 100 x100. The dispersal distance is $P = 40$. The initial species density is $I_i = 0.03$ (same for all species). The density of grazer cells is $I_y = 0.4$. Error bars indicate the standard deviations.

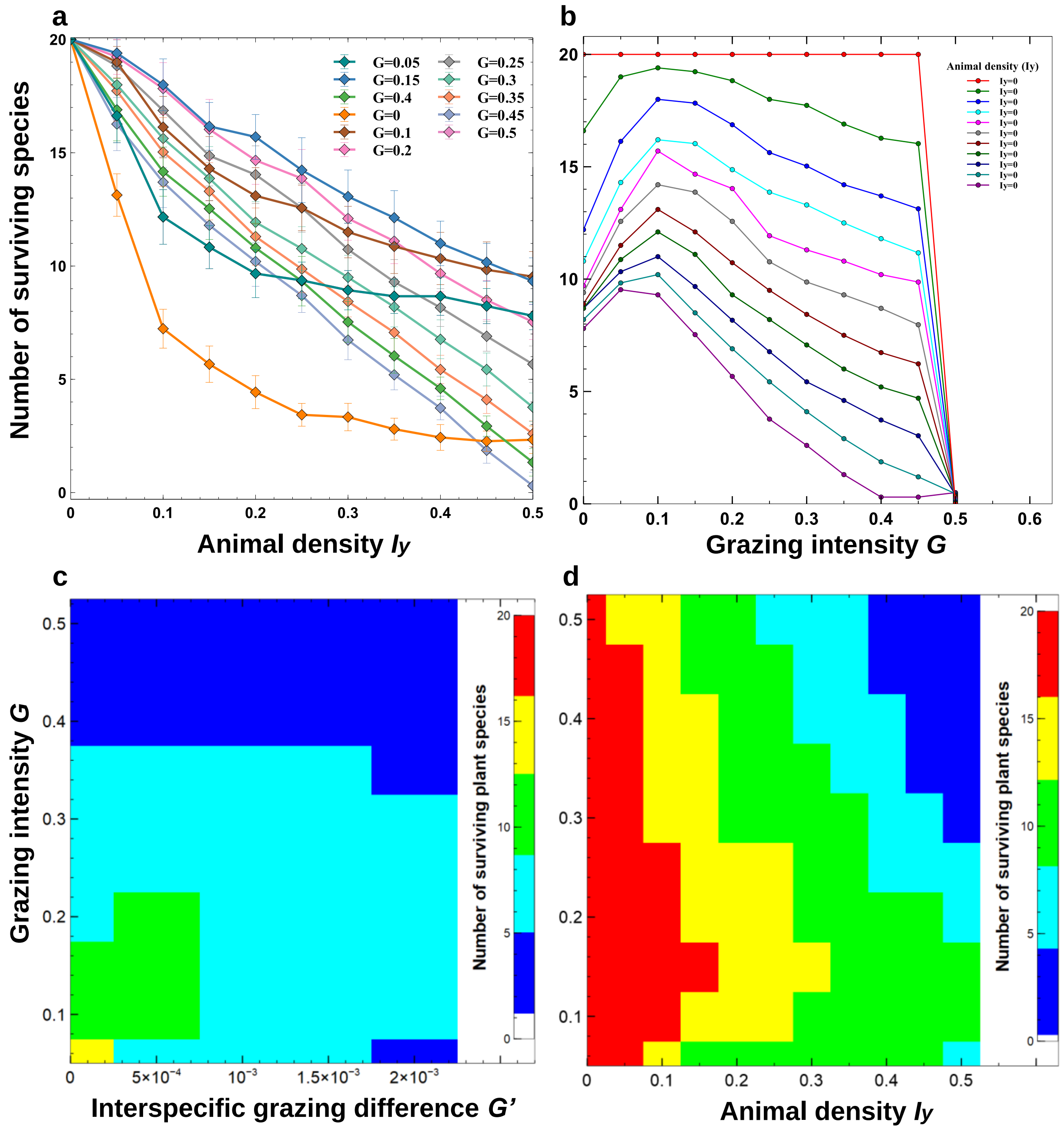

Supplementary Figure 4. The effect of the animal density I_y, basal grazing rate *G,* and interspecific grazing difference *G'* on the number of surviving species. **a-d** The grazing intensity of a species increases as the fecundity increases, i.e., $g_i = G + G'(20 - i)$ and $B_i = B$-0.002(i-1) for i=1, 2, …, s), indicating a trade-off between the birth rate and grazing intensity of a species. The lattice size is 100 x100. The dispersal distance is $P = 40$. The initial species density is $I_i =$ 0.03 (same for all species). **c** The density of grazer cells is $I_y = 0.4$. Error bars indicate the standard deviations.

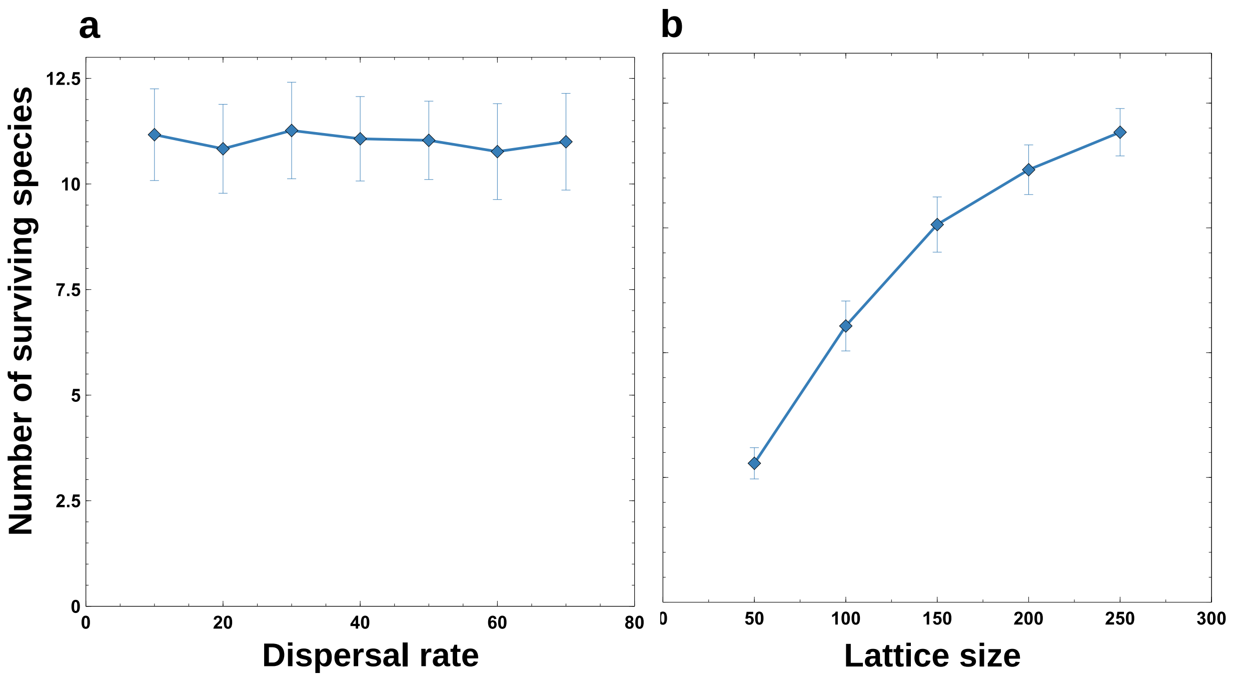

Supplementary Figure 5. The effects of dispersal range and lattice size on the number of surviving species in a grassland community under grazing. **a-b** The grazing rate of a species increases as the fecundity increases, i.e., $g_i = G + G'(20 - i)$ and $B_i = B$-0.002(i-1) for i=1, 2, …, s), indicating a trade-off between the birth rate and grazing intensity of a species. The birth rate is $B_i = B$-0.002(i-1), where B = 1. The lattice size is 100 x100 for (**a**). The dispersal distance is P = 40 for (**b**). The initial species density is I_i = 0.03 (same for all species). The density of grazer cells is I_y = 0.4. Error bars indicate the standard deviations.

Supplementary Table 1. Number of surviving species based on the grazing intensity *G* and interspecific grazing difference *G'*. The grazing intensity of a species increases as the fecundity increases, i.e., $g_i = G + G'(20 - i)$ and $B_i = B\text{-}0.002(i\text{-}1)$ for i=1, 2, …, s), indicating a trade-off between the birth rate and grazing intensity of a species. The lattice size is 100 x100. The dispersal distance is $P = 40$. The initial species density is $I_i = 0.03$ (same for all species). The density of grazer cells is $I_y = 0.4$.

**G'** \ **G**	0	0.05	0.1	0.15	0.2	0.25	0.3	0.35	0.4	0.45	0.5
0.00001	20.00	14.57	11.03	9.63	8.37	7.23	6.13	5.10	4.67	3.77	3.07
0.00002	20.00	14.07	11.37	9.50	8.60	7.37	6.50	5.20	4.17	3.90	2.93
0.00003	19.93	14.50	11.43	9.70	8.60	7.17	6.57	5.40	4.50	3.77	3.07
0.00004	18.40	15.40	11.20	9.77	8.50	7.40	6.30	5.33	4.57	3.67	3.00
0.00005	15.50	15.47	11.83	9.87	8.50	7.40	6.40	5.30	4.80	3.73	3.03
0.00006	13.20	16.10	12.20	9.87	8.40	7.57	6.40	5.57	4.47	3.60	3.07
0.00007	12.00	16.43	12.03	10.17	8.63	7.10	6.23	5.67	4.67	3.60	3.20
0.00008	10.20	16.70	12.50	10.13	8.63	7.60	6.50	5.57	4.27	3.70	3.07
0.00009	9.37	16.47	12.70	10.40	8.47	7.73	6.50	5.20	4.57	3.70	2.90
0.0001	8.73	17.03	12.43	10.33	8.73	7.50	6.03	5.40	4.67	3.77	2.93
0.0002	5.00	14.50	13.60	10.93	8.87	7.63	6.57	5.10	4.57	3.80	2.97
0.0003	3.80	11.33	13.50	11.70	9.33	7.70	6.53	5.33	4.50	3.70	3.13
0.0004	3.07	9.43	11.70	11.40	10.13	8.07	6.50	5.77	4.57	3.57	2.83
0.0005	2.53	8.37	10.40	11.17	9.67	7.83	6.87	5.43	4.47	4.07	2.80
0.0006	2.57	7.70	9.47	10.13	9.73	8.37	6.80	5.57	4.63	3.67	2.87
0.0007	2.47	7.17	8.50	9.50	9.30	8.27	6.77	5.67	4.43	3.63	2.97
0.0008	2.37	7.10	8.23	9.13	8.93	8.07	6.87	5.80	4.50	3.63	2.73
0.0009	2.17	6.53	7.67	8.47	8.30	8.03	6.87	5.67	4.73	3.63	2.73
