# Supplementary material for: Grazing enhances species diversity in grassland communities
Source: Sci Rep. 2019 Aug 1;9:11201. doi: 10.1038/s41598-019-47635-1 (PMC6671982; doi:10.1038/s41598-019-47635-1)
Supplement: Supplementary file 2 — Supplementary Information (code) [file 41598_2019_47635_MOESM2_ESM.pdf]

/***SUPPLEMENTARY MATERIALS (Simulation program**

/***Title: Grazing enhances species diversity in grassland communities**

/*Muhammad Almaududi Pulungan[1,*], Shota Suzuki[5], Maica Krizna Areja Gavina[1,2], Jerrold M. Tubay[2], Hiromu Ito[3,4], Momoka Nii[5], Genki Ichinose[1], Takuya Okabe[5], Atsushi Ishida[6], Masae Shiyomi[7], Tatsuya Togashi[9], JinYoshimura[1,8,9, *], Satoru Morita[1,*]

/*

/*

/*

/*

/*Simulation program (base program)

```
/*
/*************internal processing*************/
#include<stdio.h>
#include<stdlib.h>
#include<string.h>
#include<assert.h>
#include<math.h>
#include<time.h>
//#include<memory.h>
#define NUMBER 100
#define DISPLAY_INTERVAL 1//
#define DISPLAY_MODE 0 //

/********field parameter********/

#define O 0              //identifier
#define A 1
#define B 2
#define C 3
#define D 4
#define E 5
#define F 6
#define G 7
#define H 8
#define I 9
#define J 10
#define K 11
#define L 12
#define M 13
#define N 14
#define P 15
#define Q 16
#define R 17
#define S 18
#define T 19
#define U 20
#define Y 21
#define Nu 20
#define FILENAME "filename.csv"  //filename
```

```
double dp=0, predm=0;
double BA=0, MA=0;
double gr=0.001;
double G1=0;

#define SIZE 100
#define AREA (SIZE * SIZE)
#define outfield 10000
#define MaxStep 10000
#define trial 30
int sarray[trial];




/*************parameter*************/

//basal parameter of A
#define FdensA 0.03                    //Density of A
#define BirthA BA                      //Birth rate of A
#define MortalA 0.1                    //Mortality rate of A
#define GrazeA G1 + gr*(Nu-A)          //grazing rate A
#define DispReachA 40                  //Dispersion Reach of A

// basal parameter of A
#define FdensB 0.03                    //Density of B
#define BirthB BirthA-0.002*(B-1)      //Birth rate of B
#define MortalB 0.1                    //Mortality rate of B
#define GrazeB G1 + gr*(Nu-B)          //grazing rate B
#define DispReachB 40                  //Dispersion Reach of B

// basal parameter of C
#define FdensC 0.03                    //Density of C
#define BirthC BirthA-0.002*(C-1)      //Birth rate of C
#define MortalC 0.1                    //Mortality rate of C
#define GrazeC G1 + gr*(Nu-C)          //grazing rate
#define DispReachC 40                  //Dispersion Reach of C

// basal parameter of D
#define FdensD 0.03                    //Density of D
#define BirthD BirthA-0.002*(D-1)      //Birth rate of D
#define MortalD 0.1                    //Mortality rate of D
#define GrazeD G1 + gr*(Nu-D)          //grazing rate
#define DispReachD 40                  //Dispersion Reach of D

// basal parameter of E
#define FdensE 0.03                    //Density of E
#define BirthE BirthA-0.002*(E-1)      //Birth rate of E
#define MortalE 0.1                    //Mortality rate of E
#define GrazeE G1 + gr*(Nu-E)          //grazing rate
#define DispReachE 40                  //Dispersion Reach of E

// basal parameter of F
#define FdensF 0.03                    //Density of F
```

```
#define BirthF BirthA-0.002*(F-1)       //Birth rate of F
#define MortalF 0.1                     //Mortality rate of F
#define GrazeF G1 + gr*(Nu-F)           //grazing rate
#define DispReachF 40                   //Dispersion Reach of F

// basal parameter of G
#define FdensG 0.03                     //Density of G
#define BirthG BirthA-0.002*(G-1)       //Birth rate of G
#define MortalG 0.1                     //Mortality rate of G
#define GrazeG G1 + gr*(Nu-G)           //grazing rate
#define DispReachG 40                   //Dispersion Reach of G

// basal parameter of H
#define FdensH 0.03                     //Density of H
#define BirthH BirthA-0.002*(H-1)       //Birth rate of H
#define MortalH 0.1                     //Mortality rate of H
#define GrazeH G1 + gr*(Nu-H)           //grazing rate
#define DispReachH 40                   //Dispersion Reach of H

// basal parameter of I
#define FdensI 0.03                     //Density of I
#define BirthI BirthA-0.002*(I-1)       //Birth rate of I
#define MortalI 0.1                     //Mortality rate of I
#define GrazeI G1 + gr*(Nu-I)           //grazing rate
#define DispReachI 40                   //Dispersion Reach of I

// basal parameter of J
#define FdensJ 0.03                     //Density of J
#define BirthJ BirthA-0.002*(J-1)       //Birth rate of J
#define MortalJ 0.1                     //Mortality rate of J
#define GrazeJ G1 + gr*(Nu-J)           //grazing rate
#define DispReachJ 40                   //Dispersion Reach of J

// basal parameter of K
#define FdensK 0.03                     //Density of K
#define BirthK BirthA-0.002*(K-1)       //Birth rate of K
#define MortalK 0.1                     //Mortality rate of K
#define GrazeK G1 + gr*(Nu-K)           //grazing rate
#define DispReachK 40                   //Dispersion Reach of K

// basal parameter of L
#define FdensL 0.03                     //Density of L
#define BirthL BirthA-0.002*(L-1)       //Birth rate of L
#define MortalL 0.1                     //Mortality rate of L
#define GrazeL G1 + gr*(Nu-L)           //grazing rate
#define DispReachL 40                   //Dispersion Reach of L

// basal parameter of M
#define FdensM 0.03                     //Density of M
#define BirthM BirthA-0.002*(M-1)       //Birth rate of M
#define MortalM 0.1                     //Mortality rate of M
#define GrazeM G1 + gr*(Nu-M)           //grazing rate
#define DispReachM 40                   //Dispersion Reach of M

// basal parameter of N
```

```
#define FdensN 0.03                        //Density of N
#define BirthN BirthA-0.002*(N-1)          //Birth rate of N
#define MortalN 0.1                        //Mortality rate of N
#define GrazeN G1 + gr*(Nu-N)              //grazing rate
#define DispReachN 40                      //Dispersion Reach of N

// basal parameter of P
#define FdensP 0.03                        //Density of P
#define BirthP BirthA-0.002*(P-1)          //Birth rate of P
#define MortalP 0.1                        //Mortality rate of P
#define GrazeP G1 + gr*(Nu-P)              //grazing rate


#define DispReachP 40                      //Dispersion Reach of P

// basal parameter of Q
#define FdensQ 0.03                        //Density of Q
#define BirthQ BirthA-0.002*(Q-1)          //Birth rate of Q
#define MortalQ 0.1                        //Mortality rate of Q
#define GrazeQ G1 + gr*(Nu-Q)              //grazing rate
#define DispReachQ 40                      //Dispersion Reach of Q

// basal parameter of R
#define FdensR 0.03                        //Density of R
#define BirthR BirthA-0.002*(R-1)          //Birth rate of R
#define MortalR 0.1                        //Mortality rate of R
#define GrazeR G1 + gr*(Nu-R)              //grazing rate
#define DispReachR 40                      //Dispersion Reach of R

// basal parameter of S
#define FdensS 0.03                        //Density of S
#define BirthS BirthA-0.002*(S-1)          //Birth rate of S
#define MortalS 0.1                        //Mortality rate of S
#define GrazeS G1 + gr*(Nu-S)              //grazing rate
#define DispReachS 40                      //Dispersion Reach of S

// basal parameter of T
#define FdensT 0.03                        //Density of T
#define BirthT BirthA-0.002*(T-1)          //Birth rate of T
#define MortalT 0.1                        //Mortality rate of T
#define GrazeT G1 + gr*(Nu-T)              //grazing rate
#define DispReachT 40                      //Dispersion Reach of T

// basal parameter of U
#define FdensU 0.03                        //Density of U
#define BirthU BirthA-0.002*(U-1)          //Birth rate of U
#define MortalU 0.1                        //Mortality rate of U
#define GrazeU G1 + gr*(Nu-U)              //grazing rate
#define DispReachU 40                      //Dispersion Reach of U

// basal parameter of Y
#define FdensY     0.4                     //Density of Y
#define PredY      (1.0-predm)             //Birth rate of Y

//static double
```

```
      DensA,DensB,DensC,DensD,DensO,DensE,DensF,DensG,DensH,DensI,DensJ,DensK,DensL,
DensM,DensN,DensP,DensQ,DensR,DensS,DensT,DensU,DensY;

double birthA;            //to simulate using variable birth rate
double birthB;
double birthC;
double birthD;
double birthE;
double birthF;
double birthG;
double birthH;
double birthI;
double birthJ;
double birthK;
double birthL;
double birthM;
double birthN;
double birthP;
double birthQ;
double birthR;
double birthS;
double birthT;
double birthU;
double birthY;

int field[SIZE][SIZE];
int field2[SIZE][SIZE];

int step;

double frevisionA[SIZE][SIZE];        //field revision
double frevisionB[SIZE][SIZE];
double frevisionC[SIZE][SIZE];
double frevisionD[SIZE][SIZE];
double frevisionE[SIZE][SIZE];
double frevisionF[SIZE][SIZE];
double frevisionG[SIZE][SIZE];
double frevisionH[SIZE][SIZE];
double frevisionI[SIZE][SIZE];
double frevisionJ[SIZE][SIZE];
double frevisionK[SIZE][SIZE];
double frevisionL[SIZE][SIZE];
double frevisionM[SIZE][SIZE];
double frevisionN[SIZE][SIZE];
double frevisionP[SIZE][SIZE];
double frevisionQ[SIZE][SIZE];
double frevisionR[SIZE][SIZE];
double frevisionS[SIZE][SIZE];
double frevisionT[SIZE][SIZE];
double frevisionU[SIZE][SIZE];
double frevisionY[SIZE][SIZE];

double mrateA[SIZE][SIZE];
double mrateB[SIZE][SIZE];
double mrateC[SIZE][SIZE];
```

```
double mrateD[SIZE][SIZE];
double mrateE[SIZE][SIZE];
double mrateF[SIZE][SIZE];
double mrateG[SIZE][SIZE];
double mrateH[SIZE][SIZE];
double mrateI[SIZE][SIZE];
double mrateJ[SIZE][SIZE];
double mrateK[SIZE][SIZE];
double mrateL[SIZE][SIZE];
double mrateM[SIZE][SIZE];
double mrateN[SIZE][SIZE];
double mrateP[SIZE][SIZE];
double mrateQ[SIZE][SIZE];
double mrateR[SIZE][SIZE];
double mrateS[SIZE][SIZE];
double mrateT[SIZE][SIZE];
double mrateU[SIZE][SIZE];
double mrateY[SIZE][SIZE];

double densA[MaxStep+1];
double densB[MaxStep+1];
double densC[MaxStep+1];
double densD[MaxStep+1];
double densE[MaxStep+1];
double densF[MaxStep+1];
double densG[MaxStep+1];
double densH[MaxStep+1];
double densI[MaxStep+1];
double densJ[MaxStep+1];
double densK[MaxStep+1];
double densL[MaxStep+1];
double densM[MaxStep+1];
double densN[MaxStep+1];
double densP[MaxStep+1];
double densQ[MaxStep+1];
double densR[MaxStep+1];
double densS[MaxStep+1];
double densT[MaxStep+1];
double densU[MaxStep+1];
double densY[MaxStep+1];

/*      random number(integer)      */
int randPoint(int r){               //0 to r-1
        return rand()%r;
}
/*      random number(probability)*/
double drand48(){
        return (double)rand()/RAND_MAX;         //0 to 1
}

/*      random number initializer */
void srand48(long time_t){
        srand(time_t);
}
```

```
/*connect field ends*/
int mod(int x){
       int z;
       z = (x+SIZE)%SIZE;
       return z;
}

/* not connect field ends*/
int mod2(int x){
       int z;
       if(x>SIZE-2){
              z =outfield;
       }
       else if(x<0){

              z =outfield;

       }
       else{
              z=x;
       }
       return z;
}

/*reproduction process local*/
void ReproductionD(){                    //Reproduction Disperse
  int species;
  int rx, ry;
  int rx1,ry1;
  int r1,r2;
  double revision=0,rate=1;

intReach[31]={0,DispReachA,DispReachB,DispReachC,DispReachD,DispReachE,DispReachF,Dis
pReachG,DispReachH,DispReachI,DispReachJ,DispReachK,DispReachL,DispReachM,DispReachN,
DispReachP,DispReachQ,DispReachR,DispReachS,DispReachT,DispReachU};

  rx=randPoint(SIZE);
       ry=randPoint(SIZE);

  if(field[rx][ry]!=O){
    if(field[rx][ry]==A){
      rate=BirthA;
      species=A;
    }
    else if(field[rx][ry]==B){
      rate=BirthB;
      species=B;
    }
    else if(field[rx][ry]==C){
      rate=BirthC;
      species=C;
    }
    else if(field[rx][ry]==D){
      rate=BirthD;
      species=D;
```

```
}
else if(field[rx][ry]==E){
  rate=BirthE;
  species=E;
}
else if(field[rx][ry]==F){
  rate=BirthF;
  species=F;
}
else if(field[rx][ry]==G){
  rate=BirthG;
  species=G;
}
else if(field[rx][ry]==H){
  rate=BirthH;
  species=H;
}
else if(field[rx][ry]==I){
  rate=BirthI;
  species=I;
}
else if(field[rx][ry]==J){
  rate=BirthJ;
  species=J;
}
   else if(field[rx][ry]==K){
  rate=BirthK;
  species=K;
}
else if(field[rx][ry]==L){
  rate=BirthL;
  species=L;
}
else if(field[rx][ry]==M){
  rate=BirthM;
  species=M;
}
else if(field[rx][ry]==N){
  rate=BirthN;
  species=N;
}
else if(field[rx][ry]==P){
  rate=BirthP;
  species=P;
}
else if(field[rx][ry]==Q){
  rate=BirthQ;
  species=Q;
}
else if(field[rx][ry]==R){
  rate=BirthR;
  species=R;
}
else if(field[rx][ry]==S){
  rate=BirthS;
```

```
  species=S;
}
else if(field[rx][ry]==T){
  rate=BirthT;
  species=T;
}else if(field[rx][ry]==U){
  rate=BirthU;
  species=U;
}else if(field[rx][ry]==Y ){
}else{
  printf("something is wrong in ReproductionD1.");
}

do{
  do{
   r1=randPoint(Reach[species]*2+1)-Reach[species];
   r2=randPoint(Reach[species]*2+1)-Reach[species];
        }while(r1==0&&r2==0);
         }while(abs(r1)+abs(r2)>=Reach[species]+1);


rx1=mod(rx+r1);
ry1=mod(ry+r2);

if(field[rx][ry]==A){
  revision=frevisionA[rx1][ry1];
}
else if(field[rx][ry]==B){
  revision=frevisionB[rx1][ry1];
}
else if(field[rx][ry]==C){
  revision=frevisionC[rx1][ry1];
}
else if(field[rx][ry]==D){
  revision=frevisionD[rx1][ry1];
}
else if(field[rx][ry]==E){
  revision=frevisionE[rx1][ry1];
}
else if(field[rx][ry]==F){
  revision=frevisionF[rx1][ry1];
}
else if(field[rx][ry]==G){
  revision=frevisionG[rx1][ry1];
}
else if(field[rx][ry]==H){
  revision=frevisionH[rx1][ry1];
}
else if(field[rx][ry]==I){
  revision=frevisionI[rx1][ry1];
}
else if(field[rx][ry]==J){
  revision=frevisionJ[rx1][ry1];
}
   else if(field[rx][ry]==K){
```

```
      revision=frevisionK[rx1][ry1];
    }
    else if(field[rx][ry]==L){
      revision=frevisionL[rx1][ry1];
    }
    else if(field[rx][ry]==M){
      revision=frevisionM[rx1][ry1];
    }
    else if(field[rx][ry]==N){
      revision=frevisionN[rx1][ry1];
    }
    else if(field[rx][ry]==P){
      revision=frevisionP[rx1][ry1];
    }
    else if(field[rx][ry]==Q){
      revision=frevisionQ[rx1][ry1];
    }
    else if(field[rx][ry]==R){
      revision=frevisionR[rx1][ry1];
    }
    else if(field[rx][ry]==S){
      revision=frevisionS[rx1][ry1];
    }
    else if(field[rx][ry]==T){
      revision=frevisionT[rx1][ry1];
    }
    else if(field[rx][ry]==U){
      revision=frevisionU[rx1][ry1];
    }else if(field[rx][ry]==Y){
    }else{
      printf("something is wrong in ReproductionD2.");
    }
    /*reproduction*/
    //if(rx1!=outfield && ry1!=outfield){
    if((field[rx1][ry1]==O) && drand48()<rate*revision){
      field[rx1][ry1]=species;
    }
  }
}

/*predation process*/
void Pred(){

 int rx, ry;
 int rx2, ry2;
 int r;
 double p;

  rx=randPoint(SIZE);
  ry=randPoint(SIZE);
  p=drand48();

              if(field2[rx][ry]==Y){
                     if(field[rx][ry]!=O){
                            if(field[rx][ry]==A){
```

```
        if(p<GrazeA){
            field[rx][ry]=0;
        }
    }else if(field[rx][ry]==B){
        if(p<GrazeB){
            field[rx][ry]=0;
        }
    }else if(field[rx][ry]==C){
        if(p<GrazeC){
            field[rx][ry]=0;
        }
    }else if(field[rx][ry]==D){
        if(p<GrazeD){
            field[rx][ry]=0;
        }
    }else if(field[rx][ry]==E){
        if(p<GrazeE){
            field[rx][ry]=0;
        }
    }else if(field[rx][ry]==F){
        if(p<GrazeF){
            field[rx][ry]=0;
        }
    }else if(field[rx][ry]==G){
        if(p<GrazeG){
            field[rx][ry]=0;
        }
    }else if(field[rx][ry]==H){
        if(p<GrazeH){
            field[rx][ry]=0;
        }
    }else if(field[rx][ry]==I){
        if(p<GrazeI){
            field[rx][ry]=0;
        }
    }else if(field[rx][ry]==J){
        if(p<GrazeJ){
            field[rx][ry]=0;
        }
    }else if(field[rx][ry]==K){
        if(p<GrazeK){
            field[rx][ry]=0;
        }
    }else if(field[rx][ry]==L){
        if(p<GrazeL){
            field[rx][ry]=0;
        }
    }else if(field[rx][ry]==M){
        if(p<GrazeM){
            field[rx][ry]=0;
        }
    }else if(field[rx][ry]==N){
        if(p<GrazeN){
            field[rx][ry]=0;
        }
```

```
                                        }else if(field[rx][ry]==P){
                                                if(p<GrazeP){
                                                        field[rx][ry]=O;
                                                }
                                        }else if(field[rx][ry]==Q){
                                                if(p<GrazeQ){
                                                        field[rx][ry]=O;
                                                }
                                        }else if(field[rx][ry]==R){
                                                if(p<GrazeR){
                                                        field[rx][ry]=O;
                                                }
                                        }else if(field[rx][ry]==S){
                                                if(p<GrazeS){
                                                        field[rx][ry]=O;
                                                }
                                        }else if(field[rx][ry]==T){
                                                if(p<GrazeT){
                                                        field[rx][ry]=O;
                                                }
                                        }else if(field[rx][ry]==U){
                                                if(p<GrazeU){
                                                        field[rx][ry]=O;
                                                }
                                        }
                                }else if(field[rx][ry]==O){
                                 }
                        }else if(field2[rx][ry]!=Y){
                                }
                        }

/*mortality process of X*/
void Xmortal(){

  int rx,ry;
  double rate=0, die;

  rx=randPoint(SIZE);
  ry=randPoint(SIZE);

  if(field[rx][ry]!=O){
    if(field[rx][ry]==A){
      rate=mrateA[rx][ry];
      die=MortalA;
    }
    else if(field[rx][ry]==B){
      rate=mrateB[rx][ry];
      die=MortalB;
    }
    else if(field[rx][ry]==C){
      rate=mrateC[rx][ry];
      die=MortalC;
    }
    else if(field[rx][ry]==D){
      rate=mrateD[rx][ry];
```

```
  die=MortalD;
}
else if(field[rx][ry]==E){
  rate=mrateE[rx][ry];
  die=MortalE;
}
else if(field[rx][ry]==F){
  rate=mrateF[rx][ry];
  die=MortalF;
}
else if(field[rx][ry]==G){
  rate=mrateG[rx][ry];
  die=MortalG;
}
else if(field[rx][ry]==H){
  rate=mrateH[rx][ry];
  die=MortalH;
}
else if(field[rx][ry]==I){
  rate=mrateI[rx][ry];
  die=MortalI;
}
else if(field[rx][ry]==J){
  rate=mrateJ[rx][ry];
  die=MortalJ;
}
else if(field[rx][ry]==K){
  rate=mrateK[rx][ry];
  die=MortalK;
}
else if(field[rx][ry]==L){
  rate=mrateL[rx][ry];
  die=MortalL;
}
else if(field[rx][ry]==M){
  rate=mrateM[rx][ry];
  die=MortalM;
}
else if(field[rx][ry]==N){
  rate=mrateN[rx][ry];
  die=MortalN;
}
else if(field[rx][ry]==P){
  rate=mrateP[rx][ry];
  die=MortalP;
}
else if(field[rx][ry]==Q){
  rate=mrateQ[rx][ry];
  die=MortalQ;
}
else if(field[rx][ry]==R){
  rate=mrateR[rx][ry];
  die=MortalR;
}
else if(field[rx][ry]==S){
```

```
      rate=mrateS[rx][ry];
      die=MortalS;
    }
    else if(field[rx][ry]==T){
      rate=mrateT[rx][ry];
      die=MortalT;
    }
    else if(field[rx][ry]==U){
      rate=mrateU[rx][ry];
      die=MortalU;
    }else if(field[rx][ry]==Y){
    }else{
      printf("something is wrong in mortal.");
    }

    if(drand48()<die*rate){
      field[rx][ry]=0;
    }
  }
}

void format(){                                    //field initializer
  int i,j;
  for(i=0;i<SIZE;i++){
    for(j=0;j<SIZE;j++){
      field[i][j]=0;
      field2[i][j]=0;
      frevisionA[i][j]=1;
      frevisionB[i][j]=1;
      frevisionC[i][j]=1;
      frevisionD[i][j]=1;
      frevisionE[i][j]=1;
      frevisionF[i][j]=1;
      frevisionG[i][j]=1;
      frevisionH[i][j]=1;
      frevisionI[i][j]=1;
      frevisionJ[i][j]=1;
      frevisionK[i][j]=1;
      frevisionL[i][j]=1;
      frevisionM[i][j]=1;
      frevisionN[i][j]=1;
      frevisionP[i][j]=1;
      frevisionQ[i][j]=1;
      frevisionR[i][j]=1;
      frevisionS[i][j]=1;
      frevisionT[i][j]=1;
      frevisionU[i][j]=1;
      frevisionY[i][j]=1;

      mrateA[i][j]=1;
      mrateB[i][j]=1;
      mrateC[i][j]=1;
      mrateD[i][j]=1;
      mrateE[i][j]=1;
      mrateF[i][j]=1;
```

```
        mrateG[i][j]=1;
        mrateH[i][j]=1;
        mrateI[i][j]=1;
        mrateJ[i][j]=1;
        mrateK[i][j]=1;
        mrateL[i][j]=1;
        mrateM[i][j]=1;
        mrateN[i][j]=1;
        mrateP[i][j]=1;
        mrateQ[i][j]=1;
        mrateR[i][j]=1;
        mrateS[i][j]=1;
        mrateT[i][j]=1;
        mrateU[i][j]=1;
        mrateY[i][j]=1;
      }
    }
}

/*    disposer     */
void dispose(int species,double density){
  int num,i,j;
  int limit=(int)(AREA*density);

  for(num=0;num<limit;num++){
    do{
      i=randPoint(SIZE);
      j=randPoint(SIZE);
    }while(field[i][j]!=O);

    if(species==A){
      if(frevisionA[i][j]!=0){
       field[i][j]=species;
      }
      else if(frevisionA[i][j]==0){
       num--;
      }
    }
    else if(species==B){
      if(frevisionB[i][j]!=0){
       field[i][j]=species;
      }
      else if(frevisionB[i][j]==0){
       num--;
      }
    }
    else if(species==C){
      if(frevisionC[i][j]!=0){
       field[i][j]=species;
      }
      else if(frevisionC[i][j]==0){
       num--;
      }
    }
    else if(species==D){
```

```
    if(frevisionD[i][j]!=0){
     field[i][j]=species;
    }
    else if(frevisionD[i][j]==0){
     num--;
    }
  }
  else if(species==E){
    if(frevisionE[i][j]!=0){
     field[i][j]=species;
    }
    else if(frevisionE[i][j]==0){
     num--;
    }
  }
  else if(species==F){
    if(frevisionF[i][j]!=0){
     field[i][j]=species;
    }
    else if(frevisionF[i][j]==0){
     num--;
    }
  }
  else if(species==G){
    if(frevisionG[i][j]!=0){
     field[i][j]=species;
    }
    else if(frevisionG[i][j]==0){
     num--;
    }
  }
  else if(species==H){
    if(frevisionH[i][j]!=0){
     field[i][j]=species;
    }
    else if(frevisionH[i][j]==0){
     num--;
    }
  }
  else if(species==I){
    if(frevisionI[i][j]!=0){
     field[i][j]=species;
    }
    else if(frevisionI[i][j]==0){
     num--;
    }
  }
  else if(species==J){
    if(frevisionJ[i][j]!=0){
     field[i][j]=species;
    }
    else if(frevisionJ[i][j]==0){
     num--;
    }
  }
```

```
else if(species==K){
  if(frevisionK[i][j]!=0){
   field[i][j]=species;
  }
  else if(frevisionK[i][j]==0){
   num--;
  }
}
else if(species==L){
  if(frevisionL[i][j]!=0){
   field[i][j]=species;
  }
  else if(frevisionL[i][j]==0){
   num--;
  }
}
else if(species==M){
  if(frevisionM[i][j]!=0){
   field[i][j]=species;
  }
  else if(frevisionM[i][j]==0){
   num--;
  }
}
else if(species==N){
  if(frevisionN[i][j]!=0){
   field[i][j]=species;
  }
  else if(frevisionN[i][j]==0
){

   num--;
  }
}
else if(species==P){
  if(frevisionP[i][j]!=0){
   field[i][j]=species;
  }
  else if(frevisionP[i][j]==0){
   num--;
  }
}
else if(species==Q){
  if(frevisionQ[i][j]!=0){
   field[i][j]=species;
  }
  else if(frevisionQ[i][j]==0){
   num--;
  }
}
else if(species==R){
  if(frevisionR[i][j]!=0){
   field[i][j]=species;
  }
  else if(frevisionR[i][j]==0){
   num--;
```

```
      }
    }
    else if(species==S){
      if(frevisionS[i][j]!=0){
       field[i][j]=species;
      }
      else if(frevisionS[i][j]==0){
       num--;
      }
    }
    else if(species==T){
      if(frevisionT[i][j]!=0){
       field[i][j]=species;
      }
      else if(frevisionT[i][j]==0){
       num--;
      }
    }
    else if(species==U){
      if(frevisionU[i][j]!=0){
       field[i][j]=species;
      }
      else if(frevisionU[i][j]==0){
       num--;
      }
    }
  }
}

void Ydispose(int species,double density){
  int num,i,j;
  int limit=(int)(AREA*density);

  for(num=0;num<limit;num++){
    do{
      i=randPoint(SIZE);
      j=randPoint(SIZE);
    }while(field2[i][j]!=O);

       if(species==Y){
       if(frevisionY[i][j]!=0){
                             field2[i][j]=species;
       }
      else if(frevisionY[i][j]==0){
                      num--;
      }
    }
  }
}
void disprevC(){    //dispose revision continuous (0□`1)
  int i,j;

  for(i=0;i<SIZE;i++){
    for(j=0;j<SIZE;j++){
      frevisionA[i][j]=drand48();
```

```
      frevisionB[i][j]=drand48();
      frevisionC[i][j]=drand48();
      frevisionD[i][j]=drand48();
      frevisionE[i][j]=drand48();
      frevisionF[i][j]=drand48();
      frevisionG[i][j]=drand48();
      frevisionH[i][j]=drand48();
      frevisionI[i][j]=drand48();
      frevisionJ[i][j]=drand48();
      frevisionK[i][j]=drand48();
      frevisionL[i][j]=drand48();
      frevisionM[i][j]=drand48();
      frevisionN[i][j]=drand48();
      frevisionP[i][j]=drand48();
      frevisionQ[i][j]=drand48();
      frevisionR[i][j]=drand48();
      frevisionS[i][j]=drand48();
      frevisionT[i][j]=drand48();
      frevisionU[i][j]=drand48();
      frevisionY[i][j]=drand48();
    }
  }
}

//dispose mortality rate
  int i,j;

  for(i=0;i<SIZE;i++){
    for(j=0;j<SIZE;j++){
      mrateA[i][j]=1;
      mrateB[i][j]=1;
      mrateC[i][j]=1;
      mrateD[i][j]=1;
      mrateE[i][j]=1;
      mrateF[i][j]=1;
      mrateG[i][j]=1;
      mrateH[i][j]=1;
      mrateI[i][j]=1;
      mrateJ[i][j]=1;
      mrateK[i][j]=1;
      mrateL[i][j]=1;
      mrateM[i][j]=1;
      mrateN[i][j]=1;
      mrateP[i][j]=1;
      mrateQ[i][j]=1;
      mrateR[i][j]=1;
      mrateS[i][j]=1;
      mrateT[i][j]=1;
      mrateU[i][j]=1;
      mrateY[i][j]=1;
    }
  }
}

/*     density counter     */
```

```
double CountDensity(int target){
  int i,j;
  double number=0;
  double density=0.0;
  for(i=0;i<SIZE;i++){
    for(j=0;j<SIZE;j++){
      if(field[i][j]==target){
       number++;
      }
    }
  }
  density=number/AREA;
  return(density);
}

double CountDensity2(int target){
  int i,j;
  double number=0;
  double density=0.0;
  for(i=0;i<SIZE;i++){
    for(j=0;j<SIZE;j++){
      if(field2[i][j]==target){
       number++;
      }
    }

  }
  density=number/AREA;
  return(density);
}

int main(){
  FILE *fp;
  if ((fp = fopen(FILENAME, "w")) == NULL) {
    printf("file open error!!¥n");
    exit(EXIT_FAILURE);
  }

  srand((unsigned)time(NULL));
int nn, nn1, nn2;

      for(nn1=0,G1=0;nn1<=10;nn1++,G1+=0.05){
             for(nn2=0,BA=0;nn2<=20;nn2++,BA+=0.05){
  double s=0,a=0;
      int n=0,p=0,m=0,size=0,area;
      int k,u,v,z;

  for(p=0;p<=MaxStep;p++){       //start of density initializer
    densA[p]=0;
    densB[p]=0;
    densC[p]=0;
    densD[p]=0;
    densE[p]=0;
    densF[p]=0;
    densG[p]=0;
```

```
    densH[p]=0;
    densI[p]=0;
    densJ[p]=0;
    densK[p]=0;
    densL[p]=0;
    densM[p]=0;
    densN[p]=0;
    densP[p]=0;
    densQ[p]=0;
    densR[p]=0;
    densS[p]=0;
    densT[p]=0;
    densU[p]=0;
  }                              //end  of density initializer
float var=0;
  for(n=0;n<=trial-1;n+=1){      //start of trial

    format();

    disprevC();                  //initial disposition of fÃ

    dispose(A,FdensA);           //initial disposition of species
    dispose(B,FdensB);
    dispose(C,FdensC);
    dispose(D,FdensD);
    dispose(E,FdensE);
    dispose(F,FdensF);
    dispose(G,FdensG);
    dispose(H,FdensH);
    dispose(I,FdensI);
    dispose(J,FdensJ);
    dispose(K,FdensK);
    dispose(L,FdensL);
    dispose(M,FdensM);
    dispose(N,FdensN);
    dispose(P,FdensP);
    dispose(Q,FdensQ);
    dispose(R,FdensR);
    dispose(S,FdensS);
    dispose(T,FdensT);
    dispose(U,FdensU);

    step=0;
int sinit=s;
             for(step=0;step<=MaxStep;step++){       //start of 1 trial

                    for(u=0;u<SIZE;u++){
                     for(v=0;v<SIZE;v++){
                            field2[u][v]=O;
                     }
                    }

                    Ydispose(Y,FdensY);

                    for (m=1;m<=AREA;m++){     //size
```

```
                ReproductionD();    //reproductionprocess(local)
            for(z=0;z<3;z++){       //grazing times
                Pred();             //predation process
            }
        Xmortal;                    //natural grass mortality
           }


densA[step]=CountDensity(A);
   densB[step]=CountDensity(B);
   densC[step]=CountDensity(C);
   densD[step]=CountDensity(D);
   densE[step]=CountDensity(E);
   densF[step]=CountDensity(F);
   densG[step]=CountDensity(G);
   densH[step]=CountDensity(H);
   densI[step]=CountDensity(I);
   densJ[step]=CountDensity(J);
   densK[step]=CountDensity(K);
   densL[step]=CountDensity(L);
   densM[step]=CountDensity(M);
   densN[step]=CountDensity(N);
   densP[step]=CountDensity(P);
   densQ[step]=CountDensity(Q);
   densR[step]=CountDensity(R);
   densS[step]=CountDensity(S);
   densT[step]=CountDensity(T);
   densU[step]=CountDensity(U);


         //density output
         fprintf(fp, "%4d,", step);
         fprintf(fp, "%1.4f,", densA[step]);
         fprintf(fp, "%1.4f,", densB[step]);
         fprintf(fp, "%1.4f,", densC[step]);
         fprintf(fp, "%1.4f,", densD[step]);
         fprintf(fp, "%1.4f,", densE[step]);
         fprintf(fp, "%1.4f,", densF[step]);
         fprintf(fp, "%1.4f,", densG[step]);
         fprintf(fp, "%1.4f,", densH[step]);
         fprintf(fp, "%1.4f,", densI[step]);
         fprintf(fp, "%1.4f,", densJ[step]);
         fprintf(fp, "%1.4f,", densK[step]);
         fprintf(fp, "%1.4f,", densL[step]);
         fprintf(fp, "%1.4f,", densM[step]);
         fprintf(fp, "%1.4f,", densN[step]);
         fprintf(fp, "%1.4f,", densP[step]);
         fprintf(fp, "%1.4f,", densQ[step]);
         fprintf(fp, "%1.4f,", densR[step]);
         fprintf(fp, "%1.4f,", densS[step]);
         fprintf(fp, "%1.4f,", densT[step]);
         fprintf(fp, "%1.4f,", densU[step]);
         fprintf(fp,"¥n");

  if(step==MaxStep){                //species counter
```

```
                                        if(densA[step]>0) s++;
                                        if(densB[step]>0) s++;
                                        if(densC[step]>0) s++;
                                        if(densD[step]>0) s++;
                                        if(densE[step]>0) s++;
                                        if(densF[step]>0) s++;
                                        if(densG[step]>0) s++;
                                        if(densH[step]>0) s++;
                                        if(densI[step]>0) s++;
                                        if(densJ[step]>0) s++;
                                        if(densK[step]>0) s++;
                                        if(densL[step]>0) s++;
                                        if(densM[step]>0) s++;
                                        if(densN[step]>0) s++;
                                        if(densP[step]>0) s++;
                                        if(densQ[step]>0) s++;
                                        if(densR[step]>0) s++;
                                        if(densS[step]>0) s++;
                                        if(densT[step]>0) s++;
                                        if(densU[step]>0) s++;

    }                               //end of species counter

     printf("%d¥n",step);

    }                               //end  of 1 trial
sarray[n]=s-sinit;
  }                                 //end of trial



    fprintf(fp,"¥n");
    fprintf(fp,"Grazingrate: %f¥n Initial grazing: %f¥n Birth rate: %f¥n",gr,G1,BA );
    printf("number of survivors is %f¥n",s/trial);
    fprintf(fp,"number of survivors is %f¥n",s/trial);
    printf("lattice size is %d*%d¥n",SIZE,SIZE);
         }}
  fclose(fp);
}
```
